# High‐Specificity Spatiotemporal Cholesterol Detection by Quadrature Phase‐Shifted Polarization Stimulated Raman Imaging

**DOI:** 10.1002/anie.202505038

**Published:** 2025-06-08

**Authors:** Yongqing Zhang, Xinyu Deng, Siming Wang, Wenyu Zhou, Zhengyan Wu, Xiaobin Tang, Hyeon Jeong Lee, Delong Zhang

**Affiliations:** ^1^ Zhejiang Key Laboratory of Micro‐nano Quantum Chips and Quantum Control, School of Physics Zhejiang University Hangzhou 310027 China; ^2^ College of Biomedical Engineering & Instrument Science Zhejiang University Hangzhou 310058 China; ^3^ Key Laboratory for Biomedical Engineering of Ministry of Education Zhejiang University Hangzhou 310058 China; ^4^ MOE Frontier Science Center for Brain Science & Brain‐Machine Integration Zhejiang University Hangzhou 310058 China

**Keywords:** Cholesterol, $\chi_{1221}^{\left(3\right)}$ measurement, Molecular structural signature, Quadrature phase‐shifted polarization SRS, Real‐time imaging

## Abstract

Visualizing cholesterol dynamics in living systems in situ remains a fundamental challenge in biomedical imaging. Although fluorescence microscopy requires bulky tags that perturb small molecule behavior, stimulated Raman scattering (SRS) microscopy enables label‐free detection of CH‐rich molecules. However, conventional SRS probes only polarized Raman components, limiting molecular specificity by seemingly overlapped peaks. Here, we extend SRS microscopy to achieve rapid, comprehensive detection of Raman tensor through quadrature phase‐shifted polarization SRS (QP^2^‐SRS) microscopy. This technique exploits the underlying molecular signatures by detecting both polarized and depolarized components of third‐order nonlinear susceptibility χ^(3)^ that originates from molecular structural features. We adopt a specialized optical delay line that rapidly alternates between parallel‐ and perpendicular‐polarization states. QP^2^‐SRS enables unprecedented distinction of similar molecular species in complex mixtures, demonstrating approximately 10× enhancement in chemical specificity and 5× improvement in analytical accuracy. This enhanced sensitivity enables real‐time monitoring of lipid dynamics in living *C. elegans* and reveals component heterogeneity and morphological changes of LD in NAFLD livers. QP^2^‐SRS creates new opportunities for investigating cholesterol‐dependent biological processes in their native environment, with broad potential for chemical imaging with enhanced molecular specificity.

## Graphical abstract

Utilizing quadrature phase modulation, the phase‐shifted polarization SRS (QP^2^‐SRS) microscope enables simultaneous measurement of the ρ‐related $\chi_{1111}^{\left(3\right)}$ and $\chi_{1221}^{\left(3\right)}$ components. In cholesterol molecular measurements, QP^2^‐SRS leverages the structural differences between cholesterol and other biomolecules to enhance chemical identification and enable sensitive, real‐time cholesterol detection in living systems.

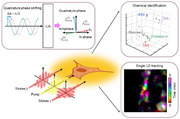

## Introduction

Cholesterol plays essential roles in cellular processes, from membrane fluidity maintenance^[^ ^1^ ^]^ to signaling molecule synthesis.^[^ ^2^ ^]^ Understanding its dynamics at single‐cell^[^ ^3^ ^]^ and tissue level^[^ ^4^ ^]^ have consistently been regarded as key to various diseases and physiological processes.^[^ ^5^ ^]^ Current analytical methods, including enzyme activity assays,^[^ ^6^ ^]^ gene expression measurements,^[^ ^7^ ^]^ and mass spectrometry,^[^ ^8^, ^9^ ^]^ can quantify cholesterol and its metabolites in bulk but lack spatial resolution. Although optical microscopy using lipid‐soluble dyes^[^ ^10^ ^]^ or fluorescence‐tagged cholesterol (e.g., filipin)^[^ ^11^ ^]^ enables visualization of intracellular cholesterol distribution, the bulky fluorescent tag inherently perturbs the behavior of these small molecules, potentially compromising their trafficking and functional roles. This limitation creates an urgent need for label‐free chemical imaging approaches to track cholesterol in complex biological systems.

Vibrational spectroscopy techniques emerge as a viable solution for this need. Based on Raman scattering and infrared (IR) absorption processes, vibrational spectroscopy leverages the unique vibrational features of molecules to provide high specificity in structural and compositional analysis, enabling precise detection and mapping of cholesterol with exceptional spatial and spectral resolution. Spontaneous Raman spectroscopy has demonstrated promise in cholesterol discrimination within model membranes^[^ ^12^ ^]^ and biological tissues.^[^ ^13^, ^14^ ^]^ Surface‐enhanced Raman scattering (SERS), using well‐designed nanoprobes, offers ultrahigh sensitivity for biosensing applications at single‐cell level.^[^ ^15^ ^]^ Alternatively, stimulated Raman scattering (SRS) signals achieve 10⁸‐fold enhancement over spontaneous Raman without external substrates,^[^ ^16^ ^]^ offering an especially promising platform for label‐free cholesterol imaging.

However, SRS microscopy still faces significant challenges in cholesterol imaging despite its advantages. Current research predominantly employs configurations with parallel‐polarized pump and Stokes fields, focusing mainly on mapping variations of the Raman cross‐section, σ. Consequently, although the spectral window is mostly adopted in the high‐wavenumber region,^[^ ^17^, ^18^, ^19^, ^20^ ^]^ overlapping vibrational bands of structurally similar biomolecules severely limit chemical specificity, particularly between cholesterol and other lipids.^[^ ^21^ ^]^ To address this limitation, various optical modulation schemes—including pulse pair‐resolved laser excitation,^[^ ^22^ ^]^ broadband excitation,^[^ ^23^, ^24^ ^]^ and spatial light modulation^[^ ^25^ ^]^—have attempted to enhance spectral information acquisition. Nevertheless, these technical refinements have not fundamentally addressed the molecular discrimination challenge. Although the fingerprint region offers rich chemical specificity, its inherently weak signal strength compromises imaging speed and sensitivity. The challenges of limitations of both chemical specificity and signal sensitivity in current SRS microscopy highlight the need for innovative approaches to overcome these constraints and achieve accurate, high‐speed cholesterol imaging.

**Figure 1:**
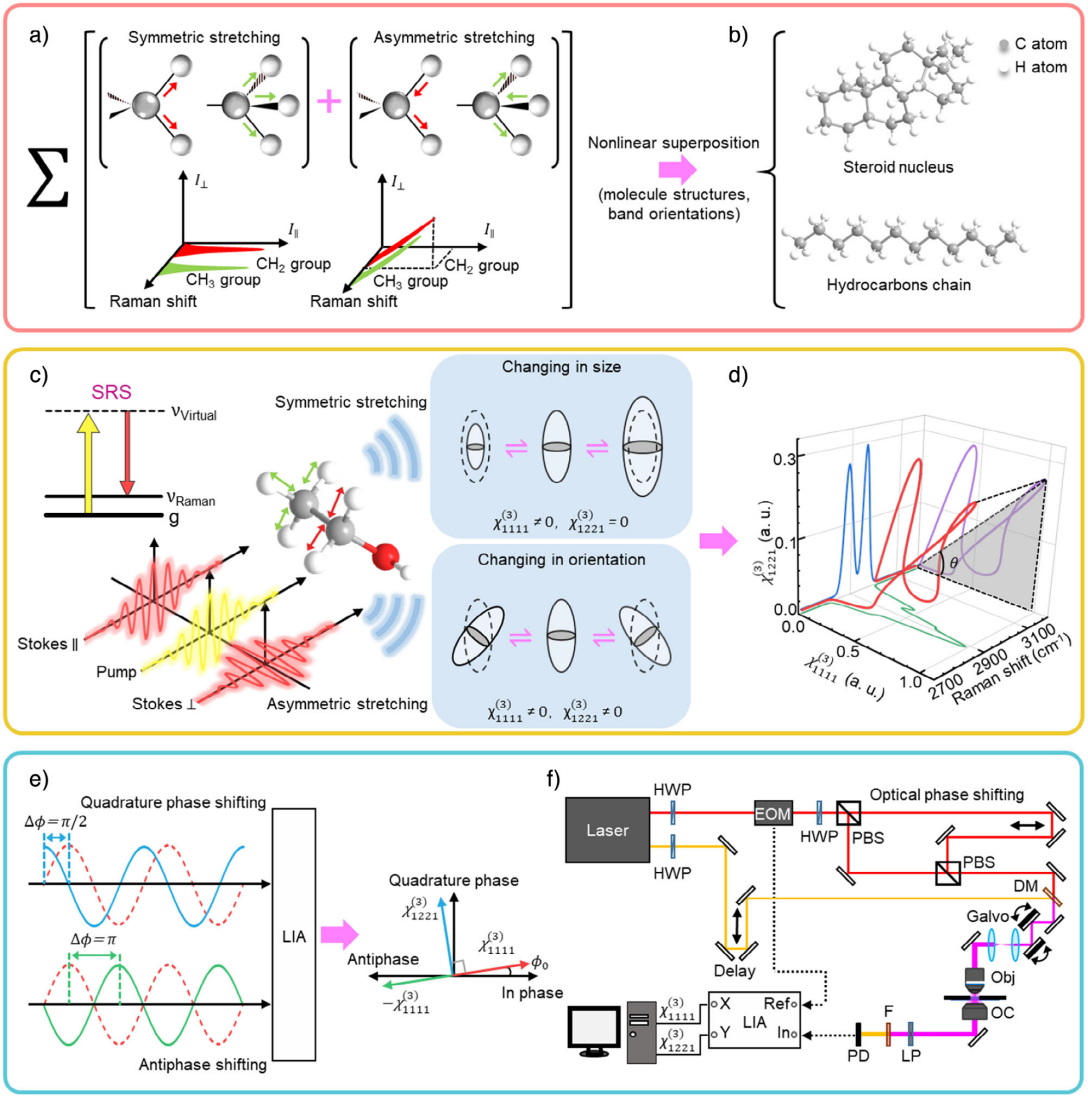
Principle of QP^2^‐SRS. a) Vibrational modes and their spontaneous Raman intensity features of CH₂ and CH₃ groups in the high‐wavenumber window. b) Different molecular structures composed by CH₂ and CH₃ groups. c) Different response types of polarizabilities excited by polarized SRS in symmetric and asymmetric stretching modes of molecules. Inset: energy diagram of SRS process. d) 3D SRS spectra including perpendicular ($\chi_{1221}^{\left(3\right)}$) and parallel ($\chi_{1111}^{\left(3\right)}$) information. The 3D spectral curve was obtained from peak fitting results of polypropylene spectra. e) Optical phase shifting with different phase differences and signal demodulation of different phase shifting in channels of LIA. ϕ_0_ is the initial phase of reference frequency. f) QP^2^‐SRS imaging system setup. HWP, half‐wave plate; EOM, electro‐optic modulator; PBS, polarizing beam splitter; Obj, objective; OC, oil condenser; LP, linear polarizer; F, filter; PD, photodiode; LIA, lock‐in amplifier.

Here, we demonstrate a new approach that breaks through these limitations by exploiting the underlying physics of molecular vibrations (Figure 1a,b). We introduce quadrature phase‐shifted polarization SRS (QP^2^‐SRS) microscopy, which achieves comprehensive detection of Raman tensors by simultaneously capturing both polarized and depolarized components of third‐order nonlinear susceptibility χ^(3)^ (Figure 1c,d). Utilizing structural and orientational variations of molecules, this approach captures different polarization responses to enhance spectral distinctions without the need for intricate optical path configurations. This advanced technique achieves precise determination of χ^(3)^ components by implementing temporal control of differently polarized Stokes beams. It offers a remarkably simple yet effective solution for enhanced molecular specificity and sensitivity, particularly for distinguishing cholesterol from other lipids in complex biological environments. Its versatility and precision make it applicable across cellular, tissue, and organismal levels, providing a powerful tool for high‐resolution molecular imaging.

## Results and Discussion

### Quadrature Phase Modulation in QP^2^‐SRS

The key implementation of QP^2^‐SRS is the quadrature phase modulation, which enables simultaneous detection of orthogonal polarization states, implemented by beam splitting and spatial offset. This approach creates a π/2 phase difference between parallel and perpendicular polarization components, enabling the detection of two types of molecular vibrational spectral information using a single photodetector (Figure 1e,f, Δϕ  =  π/2). Unlike beam‐splitting techniques used in other approaches for doubling repetition frequency,^[^ ^26^ ^]^ where uneven spacing will introduce background interference, our method just results in a phase deviation of signal from π/2 under uneven spacing conditions. This phase shift can be effectively corrected through non π/2 phase‐sensitive detection in the lock‐in amplifier (LIA), ensuring the retrieval of a clean signal without background contribution.^[^ ^27^ ^]^ Compared to existing π‐phase modulation scheme to perform a subtracting operation (Figure 1e, Δϕ  =  π), mostly for background removal,^[^ ^28^ ^]^ quadrature phase modulation is capable of a comprehensive Raman tensor measurement by simultaneously detecting two χ^(3)^ components in orthogonal channels, facilitating the distinction of structure related features (Figure 1e, Δϕ  =  π/2).

Notably, the two components χ^(3)^ associated with ρ, extracted via this polarization modulation technique, are theoretically equivalent to the information obtained using rotating half‐wave plates in conventional polarized SRS microscopy. However, optical phase shifting approach in QP^2^‐SRS offers unique advantages, including improved resistance to long‐term noises such as pump beam power fluctuations and sample motion artifacts. Additionally, SRS signals generate on a picosecond timescale. By temporally separating two signals with a quarter cycle (12.5 ns in our system), we effectively mitigate potential polarization distortions. This separation prevents background artifacts and spectral distortion. QP^2^‐SRS allows rapid switching between parallel‐ and perpendicular‐polarization SRS imaging at 20 MHz and support simultaneous dual‐polarization imaging with high spatial resolution (Figures 1f and S1). This is achieved through a combination of half‐wave plate rotation and precise phase‐shifting control of the polarized Stokes beams (Experimental methods in Supporting Information). The optical setup demonstrates minimal crosstalk between polarization channels (Figure S2), ensuring reliable and accurate imaging results in biological systems.

### High‐Specificity and High‐Accuracy Cholesterol Identification

**Figure 2:**
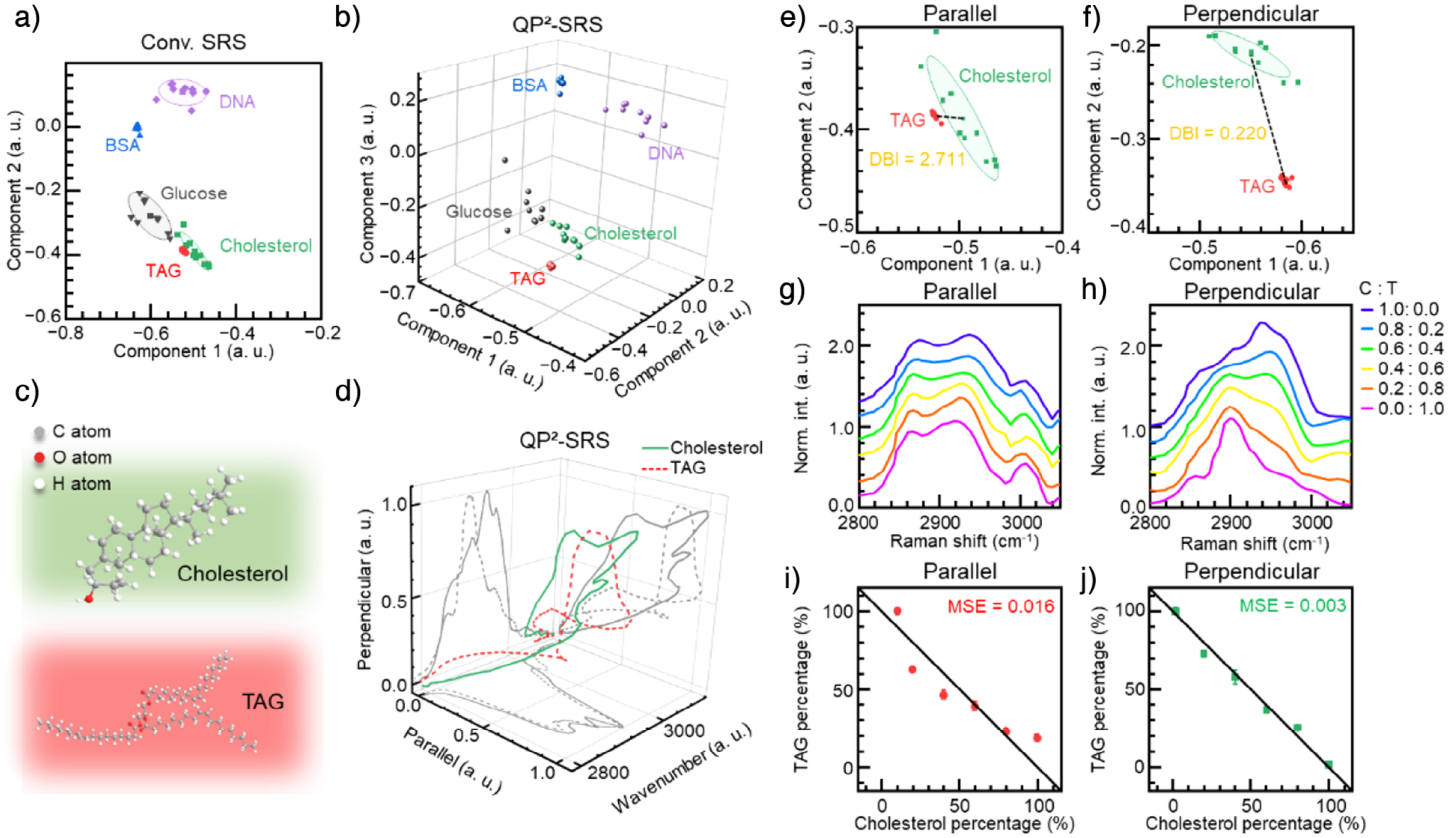
High‐specificity and high‐accuracy imaging capability of QP^2^‐SRS microscopy. a) Phasor plots of SRS spectra of biomolecules in conventional SRS mode. b) Phasor plots of SRS spectra of biomolecules in QP^2^‐SRS mode. c) Molecular structures of cholesterol and TAG. d) QP^2^‐SRS spectra of cholesterol and TAG. Phasor plots of QP^2^‐SRS spectra of cholesterol and TAG in e) parallel and f) perpendicular configurations in QP^2^‐SRS. The solid points stand for cluster centroids. Black dashed lines stand for the distances between two inter‐cluster centroids in two configurations. The ellipses represent the average confidence level of cluster centroids. DBI, Davies–Bouldin index. Spectra changing of mixed solutions in g) parallel and h) perpendicular configurations in QP^2^‐SRS. Concentrations of two components in experiment versus standard curve in i) parallel and j) perpendicular configurations in QP^2^‐SRS. Note that the conventional SRS imaging is equivalent to QP^2^‐SRS imaging at parallel configuration. Standard curve is *y*  =   − *x* + 1. MSE, mean square error.

To evaluate chemical specificity, we compared the discrimination capability of conventional SRS versus QP^2^‐SRS for various biomolecules (Figure 2a,b). The incorporation of perpendicular polarization information in QP^2^‐SRS remarkably enhanced its chemical specificity, as evidenced by its superior performance in distinguishing individual molecular species (Figure 2b and Video S1), suggesting broad applicability in biomolecular imaging and analysis. Additional evidence from QP^2^‐SRS spectra of polymers revealed distinctive peaks that remained unintelligible in conventional SRS measurements, further demonstrating QP^2^‐SRS's superior capability through spectral tensor detection (Figure S3).

Notably, QP^2^‐SRS effectively highlighted CH₃ asymmetric vibration in cholesterol (Figure 2c), revealing clear spectral differences from TAG in $\chi_{1221}^{\left(3\right)}$ measurement (Figure 2d). Quantitative spectral phasor analysis further quantified discrimination capability using the Davies–Bouldin index (Experimental methods in Supporting Information), demonstrating that QP^2^‐SRS in perpendicular configuration provides approximately 10× improvement in cholesterol‐TAG discrimination compared to conventional SRS (Figure 2e,f). To further validate this specificity, we examined a diverse array of lipid species, confirming the robust performance of QP^2^‐SRS in complex lipidomic environments (Figure S4A,B). Importantly, results from structurally similar sterols highlight that QP^2^‐SRS reliably tracks the steroid backbone and the potential for sterols identification (Figure S4C,D).

To demonstrate quantitative accuracy, we performed spectral unmixing analysis on mixed solutions containing varying proportions of these lipid components in both imaging configurations (Figure 2g,h). The unmixing results revealed that QP^2^‐SRS provides approximately 5× improvement in quantitative accuracy for cholesterol detection (MSE = 0.003) compared to conventional SRS (MSE = 0.016) when compared to standard curves (Figure 2i,j).

To assess the improvement from conventional SRS fingerprint region imaging (known for significantly weaker signals although higher specificity due to distinct peaks), we compared QP^2^‐SRS with conventional SRS on olive oil samples. For the C─H bond in ─CH₂ structure, QP^2^‐SRS imaging in the high‐wavenumber region achieves approximately 6× improvement in signal‐to‐background ratio compared to conventional fingerprint‐region SRS imaging without requiring extensive wavelength tuning (Figure S5). Additionally, QP^2^‐SRS demonstrated a 3× lower limit of detection for cholesterol at 2960 cm^−1^, reflecting enhanced sensitivity through improved spectral specificity (Figure S6). These findings establish QP^2^‐SRS microscopy as a powerful approach for high‐specificity, high‐accuracy cholesterol identification in biological systems by effectively capturing unique structural features.

### High‐Selectivity Cholesterol Imaging in HEK Cells

To evaluate the selectivity of QP^2^‐SRS microscopy in detecting cholesterol, we conducted cellular cholesterol distribution studies. We performed cholesterol depletion experiments using methyl‐β‐cyclodextrin (MβCD), a widely established compound for selectively extracting cellular cholesterol from membranes. Following established protocols (Experimental methods in Supporting Information),^[^ ^29^, ^30^, ^31^ ^]^ we treated HEK cells with MβCD and performed hyperspectral QP^2^‐SRS imaging on both treated and untreated control cells. Component analysis based on the full hyperspectral imaging dataset using LASSO algorithm showed significantly reduced cholesterol content in HEK cells after MβCD treated (Figure S7, *p* = 0.0008 for cholesterol), whereas other cellular components remained largely unchanged. This selective reduction in cholesterol signal provides strong experimental evidence for the specificity of our QP^2^‐SRS approach in complex biological systems.

**Figure 3:**
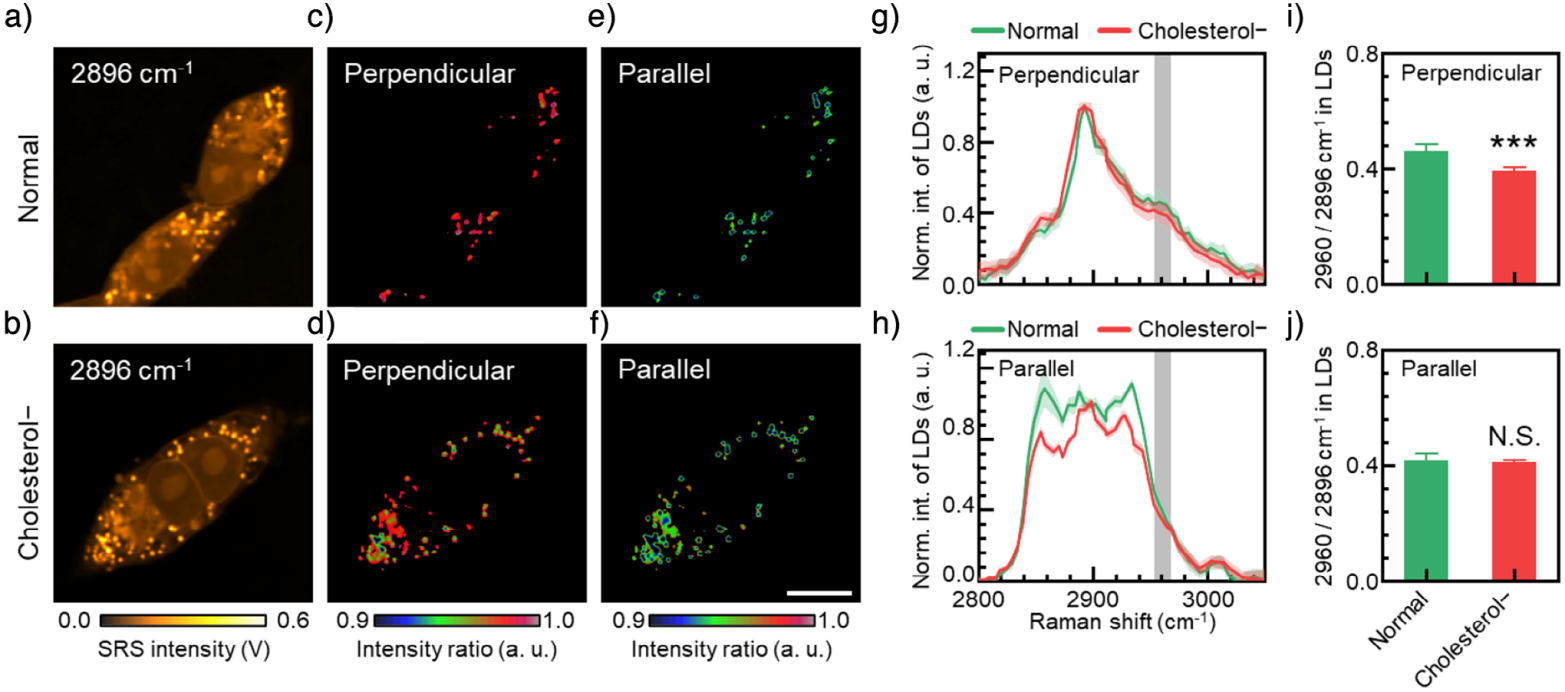
High‐selectivity imaging of HEK cells. a) and b) QP^2^‐SRS imaging of HEK cells at 2896 cm^−1^ in conventional SRS. 2960 cm^−1^/2896 cm^−1^ ratiometric images of LDs in HEK cells in c) and d) perpendicular and e) and f) parallel configurations in QP^2^‐SRS. g) and h) Average SRS spectra of LDs extracted from HEK cells (*n* = 18 for normal group, *n* = 22 for cholesterol − group). i) and j) Intensity changes at 2960 cm^−1^ in QP^2^‐SRS imaging. *, *p* < 0.05; **, *p* < 0.01; ***, *p* < 0.001; N.S., no significance. Error bars stand for +1.5 SEM. Scale bars stand for 10 µm.

To further enhance imaging efficiency and throughput for practical applications, we implemented dual‐wavelength ratiometric QP^2^‐SRS imaging to monitor changes in cholesterol content. Following cholesterol depletion using MβCD in the cells, we performed ratiometric imaging of lipid droplets (LDs) in HEK cells at 2960 cm^−1^ (characteristic of cholesterol) and 2896 cm^−1^ (general lipid content) (Figures 3a–f and S8). QP^2^‐SRS imaging clearly identified compositional differences that remained undetectable by conventional SRS method, revealing a significant decrease in cholesterol content with *p* < 0.001 (Figure 3g–j). The inability of conventional SRS imaging to detect these changes can be attributed to overlapping spectral features and limited spectral discrimination capability, which obscure subtle variations in cholesterol content and lead to substantial intergroup uncertainties in measurements (Figure 3g,h).

In summary, QP^2^‐SRS imaging offers enhanced selectivity for molecular detection, eliminating the need for complex analytical methods or time‐consuming spectral acquisition, thus providing a robust and efficient approach for cholesterol detection in cells.

### High‐Precision Localization of Metabolites

**Figure 4:**
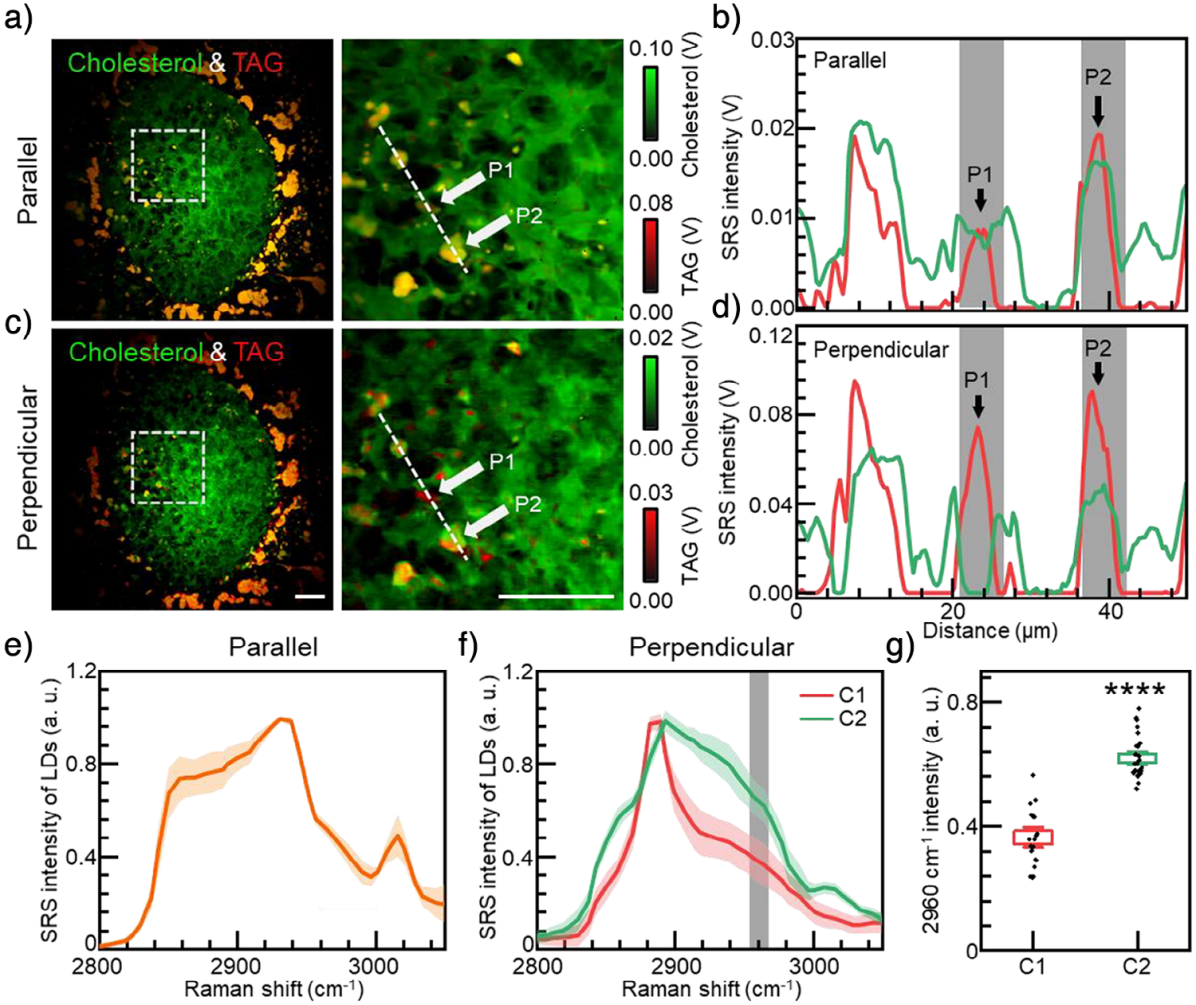
High‐precision localization of different biomolecules in QP^2^‐SRS imaging. a) Merge images of cholesterol and TAG channels in parallel configuration imaging. Zoomed‐in view indicated by dashed box in left images. b) Profiles of different LDs in parallel configuration indicated by dashed lines in (a). Arrows indicated different LDs. c) Merge images of cholesterol and TAG channels in perpendicular configuration by QP^2^‐SRS imaging. Zoomed‐in view indicated by dashed box in left images. d) Profiles of different LDs in perpendicular configuration indicated by dashed lines in (c). Arrows indicated different LDs. e) and f) Average LDs spectra obtained by LASSO algorithm. g) Statistical intensity of different LDs spectra at 2960 cm^−1^ in QP^2^‐SRS shadows in (f). (*n* = 18 for C1 group, *n* = 26 for C2 group). *, *p* < 0.05; **, *p* < 0.01; ***, *p* < 0.001, ****, *p* < 0.0001. Error bars stands for ±1.5 SEM. Scale bars stand for 25 µm.

The spatial distribution of sterols in biosystems is crucial for maintaining proper biological function and regulating metabolic pathways.^[^ ^32^, ^33^ ^]^ Here, we imaged lipid‐rich structures in seminiferous tubules of mice to showcase the capability of QP^2^‐SRS microscopy in metabolite localization. Utilizing LASSO combined with the BM4D denoising algorithm,^[^ ^17^ ^]^ effective separation of cholesterol and TAG components was achieved in both conventional SRS and QP^2^‐SRS imaging in perpendicular configuration (Figures 4a–d and S9A,B). The results of cholesterol separation by LASSO were well aligned with the localization results of cholesterol labeled by filipin (Figure S10), demonstrating the effectiveness and reliability of QP^2^‐SRS strategy. In conventional SRS, all LDs appeared to contain approximately equal amounts of TAG and cholesterol (Figure 4a,b). However, QP^2^‐SRS imaging revealed significant compositional heterogeneity between different LDs (Figure 4c,d), which was further demonstrated by distinct spectral profiles observed in these LD regions (Figure S9C,D). Comprehensive analysis of spectral data across all LD distributions indicated that although conventional SRS imaging suggested a homogeneous composition of LDs (Figure 4e), QP^2^‐SRS microscopy enabled precise differentiation of LD subpopulations, exhibiting statistically significant compositional differences (*p* < 0.0001) at 2960 cm^−1^ (Figure 4f,g).

Furthermore, the characteristics of LD compositions across entire seminiferous tubule sections were accessed by QP^2^‐SRS imaging (Figure S11A,B). We observed distinct spatial patterns in LD distribution: TAG‐dominant LDs were predominantly located in the central region of the seminiferous tubules (Figure S11C–E, ROI1), whereas clusters of densely packed, cholesterol‐rich LDs were concentrated at the periphery (Figure S11C–E, ROI2). This unique spatial distribution of lipids within seminiferous tubules provides valuable insights into lipid metabolism and its role in supporting tissue function during spermatogenesis. In summary, high molecular specificity provided by QP^2^‐SRS imaging has revealed LD compositional diversity and spatiotemporal dynamics, advancing our understanding of lipid metabolism in complex tissues.

### Lipid Metabolism Analysis in NAFLD

Neutral lipids and LDs play critical roles in regulating cellular functions and maintaining lipid homeostasis. However, our understanding of neutral lipid metabolism and its contribution to tissue function remains limited.^[^ ^34^, ^35^ ^]^ Lipotoxicity, characterized by excessive lipid accumulation, is a key factor in nonalcoholic fatty liver disease (NAFLD) as it can trigger or worsen inflammation and fibrosis associated with nonalcoholic steatohepatitis (NASH).^[^ ^36^, ^37^ ^]^ Here, we investigated alterations in lipid metabolism of mouse liver tissues during the progression of NAFLD.

**Figure 5:**
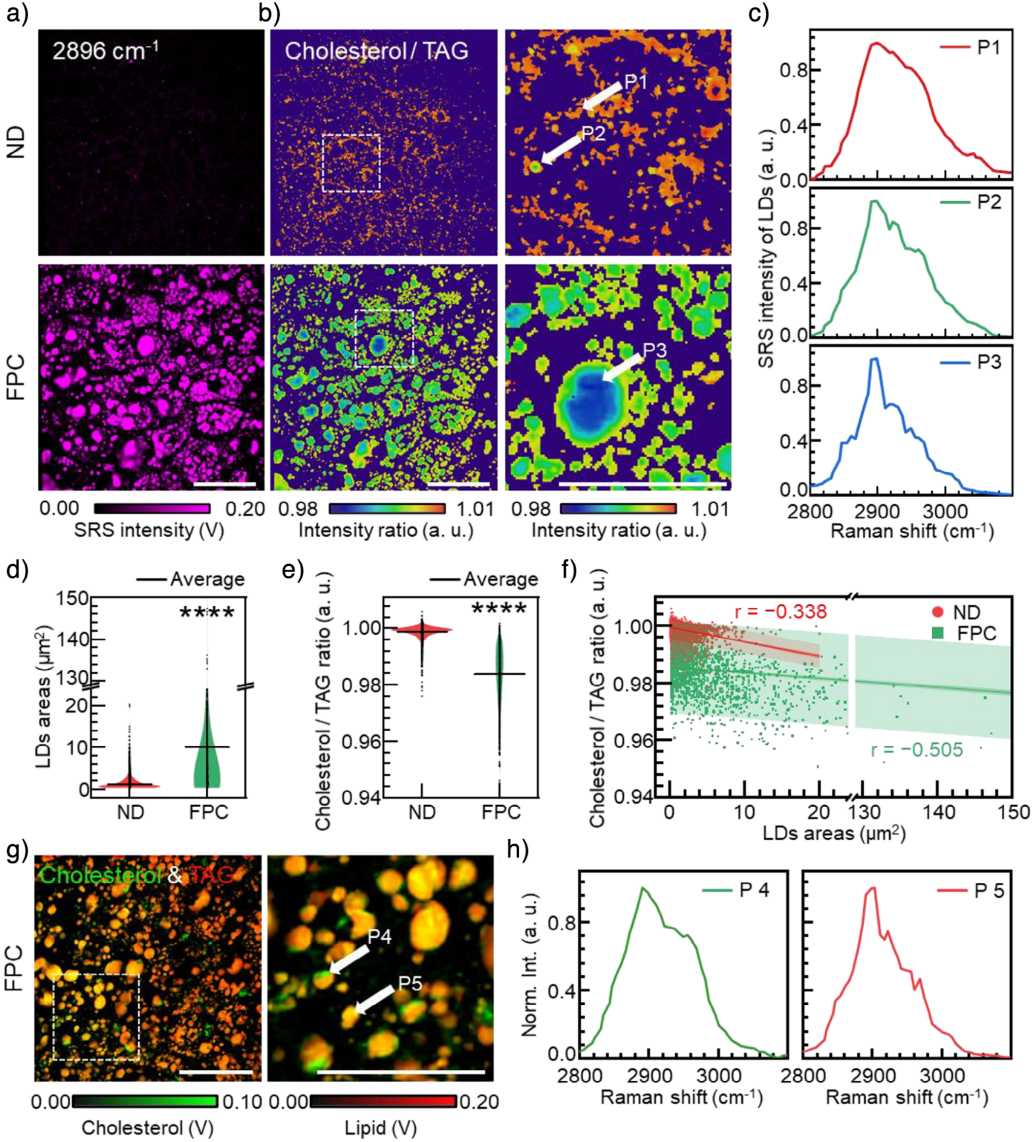
Lipid metabolism analysis of liver tissue in NAFLD mice. a) QP^2^‐SRS imaging at 2896 cm^−1^ of liver tissues in 32‐week‐old mice of ND and FPC group. b) Spatial lipid component distribution mapping obtained by LASSO analysis of liver tissues and zoomed‐in views indicated by white dashed boxes. c) QP^2^‐SRS spectra of different types of LDs indicated by arrows in (b). d) Average area of LDs changing in ND and FPC groups. e) Lipid component analysis of LDs in different groups of liver tissues. f) Correlation between size of LD aggregates and cholesterol/TAG ratio of different groups. g) Merge image and zoomed‐in view of TAG and cholesterol components of LDs in FPC mice liver. h) SRS spectra of different locations of LDs indicated by arrows in (d). *, *p* < 0.05; **, *p* < 0.01; ***, *p* < 0.001; ****, *p* < 0.0001. *n* = 3 for both groups. Scale bars stand for 50 µm.

Using QP^2^‐SRS imaging and LASSO analysis, we mapped lipid distribution in liver tissues of 32‐week‐old mice on a normal diet (ND) and a fructose, palmitate, cholesterol, and trans‐fat (FPC) diet to study NAFLD‐related lipid changes (Figures 5a–c and S12). After extracting LDs from nonlipid backgrounds, the distribution maps of TAG and cholesterol (Figure S12A) along with ratiometric images and spectral profiles (Figure 5b,c) revealed that FPC‐diet mice exhibited notable accumulation of LDs, about 8× larger than that in healthy mice (Figure 5d, *p* < 0.0001), in which major compositions are TAG (Figure 5e, *p* < 0.0001). In contrast, LDs in ND group were characterized by a higher relative cholesterol content compared to those in FPC‐diet livers (Figure 5e). Importantly, in both groups, correlations were observed between lipid composition changes and LD sizes: the larger the LDs, the lower the cholesterol content (Figure 5f). Similar trends were observed in 16‐week‐old mice fed a high‐fat diet (HFD), which exhibited significant increases in LD size and concomitant reductions in cholesterol levels compared to control groups (Figure S12B–G). These observations suggest that the reduction in cholesterol content and the enlargement of LDs are features of NAFLD progression, manifesting similarly across both the initial and advanced phases of the disease pathogenesis.

Furthermore, hyperspectral QP^2^‐SRS imaging revealed a heterogeneous distribution of cholesterol within abnormally large LDs in the liver tissues of FPC mice (Figure 5g,h), which could serve as a signature of NAFLD progression.^[^ ^38^ ^]^ This abnormal distribution may serve as both a disease progression marker and a driver of inflammation,^[^ ^39^ ^]^ aligning with prior findings on cholesterol‐induced inflammasome activation.^[^ ^40^ ^]^ If the mechanistic link between cholesterol distribution heterogeneity and inflammatory activation can be fully elucidated, future diagnostic strategies, such as QP^2^‐SRS‐based imaging, may enable early identification of high‐risk patients by detecting specific cholesterol patterns or lipid compositional signatures in hepatocytes. In summary, although further mechanistic studies are needed, these insights into changes in lipid composition, distribution, and LD morphology, which collectively contribute to the future development of this disease pathophysiology.

### Real‐Time Tracking in Living Organisms

**Figure 6:**
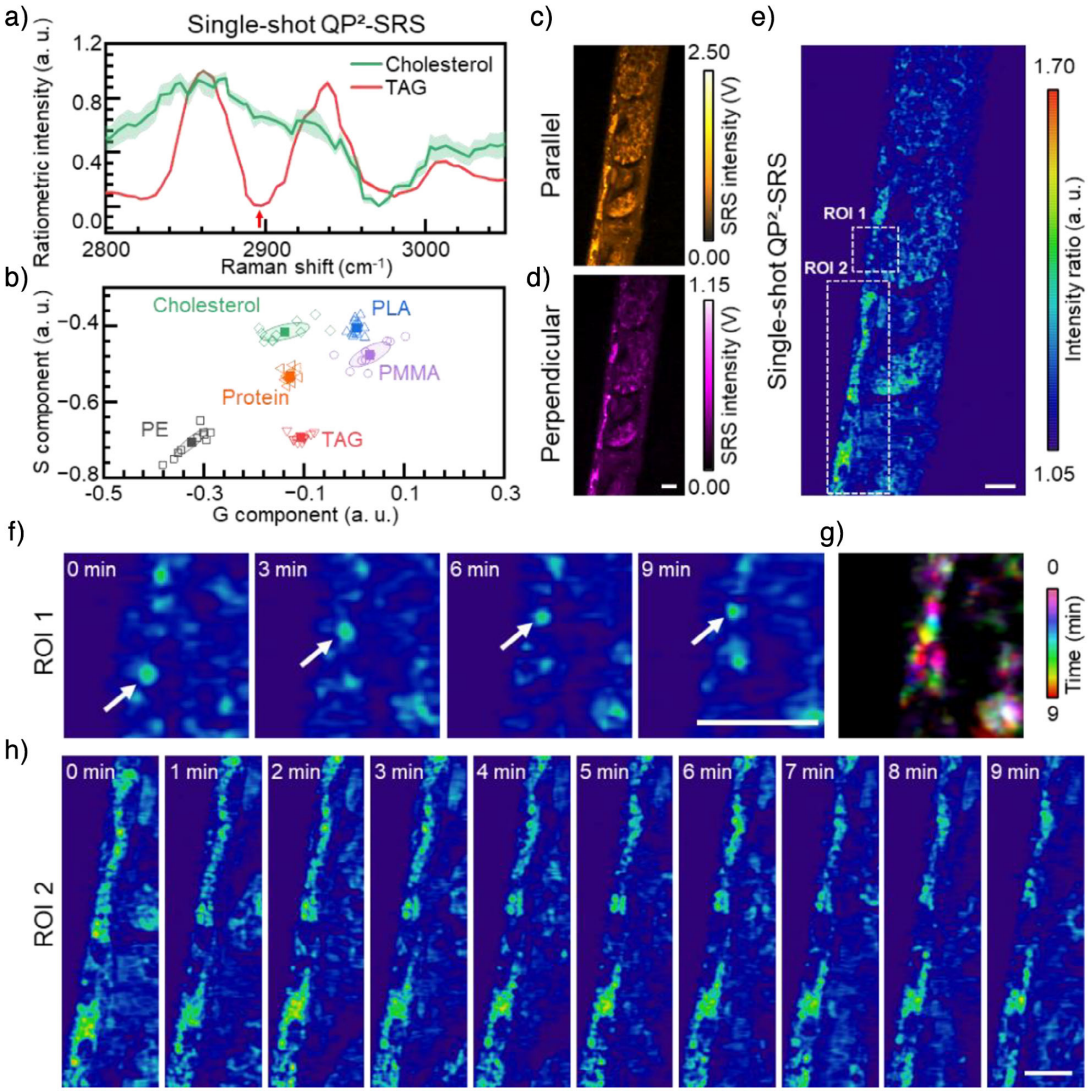
Real‐time dual‐polarization imaging of cholesterol in living *C. elegans*. a) Ratiometric sepctra of cholesterol and TAG in single‐shot QP^2^‐SRS microscopy. b) Phasor plots of QP^2^‐SRS spectra of six different molecules in single‐shot QP^2^‐SRS. c) and d) QP^2^‐SRS imaging of lipid structures at 2896 cm^−1^ of living *C. elegans*. e) Single‐shot SRS ratiometric image of *C. elegans* obtained from (a). f) Ratiometric QP^2^‐SRS imaging of LDs in *C. elegans* indicated by dashed box of ROI 1 in (e) at different times. g) Dynamic imaging of a single LD indicated by arrows in (f). h) Time‐lapse single‐shot QP^2^‐SRS imaging of LDs clustering in *C. elegans*, indicated by dashed box of ROI 2 in e). Scale bars stand for 20 µm.

For high‐throughput and cholesterol dynamics detection, we developed single‐shot QP^2^‐SRS by simultaneous acquisition of dual polarization information. Ratiometric spectra of TAG and cholesterol in QP^2^‐SRS (Figure 6a) highlighted the significance of 2896 cm^−1^ Raman shift in distinguishing cholesterol from TAG. This spectral feature enables real‐time tracking of TAG and cholesterol components within LDs using single‐shot QP^2^‐SRS imaging. Comprehensive spectral data demonstrated the chemical specificity for discrimination different components in complex mixtures (Figure 6b). Consequently, using single‐shot QP^2^‐SRS, we imaged lipid‐rich regions in seminiferous tubules at 2896 cm^−1^ (Figure S13A) and generated ratiometric images (Figure S13B). The analysis revealed lipid substructures with varying cholesterol‐to‐TAG ratios, visually represented as a gradient from high (red) to low (blue) cholesterol content in the ratiometric image (Figure S13B). The spectral profiles also demonstrated changes in the proportions of TAG and cholesterol (Figure S13C,D).

Furthermore, we demonstrate in vivo, real‐time tracking of lipid dynamics in living *Caenorhabditis elegans* (*C. elegans*) and successfully mapped cholesterol distribution in LDs at 2896 cm^−1^ using single‐shot QP^2^‐SRS imaging (Figure 6c–e). Continuous acquisition with 6‐s intervals over periods extending to 9 min revealed two distinct behavioral patterns of LDs within the epithelium of *C. elegans*. First, QP^2^‐SRS captured the directional motion of a single LD, as shown in Figure 6f and Video S2. The dynamic trajectory of this LD is represented in Figure 6g as a color‐coded image. Unlike the random Brownian motion, this LD exhibited purposeful upward movement, probably suggesting its involvement in lipid transport processes. Additionally, we discovered another phenomenon of different LDs’ clustering and components changing during 9 min (Figure 6h and Video S3). These lipid dynamics, both spatially and in terms of compositional alterations, highlight the complexity of lipid distribution and potential metabolic activities. Overall, due to its high stability and accuracy, single‐shot QP^2^‐SRS imaging holds broad applicability across diverse research fields.

## Conclusion

Unlike relying solely on Raman polarizability tensor α in different vibrational modes as the basis for molecular specificity, QP^2^‐SRS surpasses conventional SRS and SERS by integrating direct vibrational‐level matching with molecule‐specific Raman features derived from molecular structure, which enables Raman spectral analysis with improved specificity and maintains the advantage of requiring no substrate modification. By employing quadrature phase modulation, QP^2^‐SRS microscope offers a significant advancement in spectroscopic imaging by doubling the spectral information and extending it into an additional dimension, $\chi_{1221}^{\left(3\right)}$. Similar to recent innovations in combining IR and Raman spectral dimensions,^[^ ^41^ ^]^ this approach highlights the capability of multispectral dimensional imaging to extract more comprehensive molecular information from complex biological environments. Notably, QP^2^‐SRS technology can be extended to various biomolecules with distinct carbon‐chain structures (e.g., nucleic acids, carbohydrates, and lipids). Its multidimensional spectral analysis capability significantly enhances molecular identification accuracy in complex metabolic studies. This approach not only provides novel insights into key challenges such as lipid chain‐length determination and real‐time discrimination of unsaturated lipids but also opens avenues for systematically investigating structure–function relationships in biomolecules.

The advantages of QP^2^‐SRS extend beyond enhanced specificity, as the technique effectively harnesses complementary strengths of both polarization configurations. While perpendicular configuration improves molecular specificity, parallel configuration in QP^2^‐SRS retains superior signal intensity and preserves the spectral features of symmetric vibrational modes.^[^ ^42^ ^]^ By synergistically combining molecular symmetry information with absolute intensity of SRS signals, QP^2^‐SRS harnesses enhanced capabilities for vibrational imaging in complex biological environments.

Our system and method have broad applicability. For the orientation of macrostructures and local molecular alignments, QP^2^‐SRS microscopy can meet the need for depolarization ratio information extraction, which holds significant value for studying molecular symmetry and has important applications in dynamic chemical detection. Additionally, this imaging system can simultaneously capture other χ^(3)^ components by inducing circular polarization modulation, showing potential as an important platform for studying additional chemical selectivity information.^[^ ^43^, ^44^ ^]^ Not limited to the high‐wavenumber region, similar to vibrational circular dichroism,^[^ ^45^ ^]^ Raman optical activity is also widely applied in fields such as biomolecular structure.^[^ ^46^ ^]^ It is possible to achieve collection of left‐ and right‐circularly SRS spectra by modifying the imaging optical path. The concept of polarization imaging can also be extended to other optical systems. Mid‐infrared absorption spectroscopy can be used to extract polarization information from ordered structural samples that have important applications in identifying the vibrational bands.^[^ ^47^ ^]^

Future development involves the following aspects. The signal‐to‐noise ratio of QP^2^‐SRS imaging can be further improved by using balanced detection^[^ ^48^ ^]^ and quantum‐enhanced technology,^[^ ^49^, ^50^ ^]^ improving image quality and laying the foundation for more precise spectral analysis. To probe different modes simultaneously, dual‐wavenumber perpendicular polarization can be achieved using a femtosecond laser system combined with spectral focusing geometry. This not only improves hyperspectral imaging speed but also enhances the ability to capture dynamic processes.^[^ ^51^ ^]^ Additionally, the application of T‐SRS technology^[^ ^52^ ^]^ or low‐temperature Raman spectroscopy^[^ ^53^, ^54^ ^]^ can effectively near the natural linewidth limit of Raman bands, thereby further improving the resolution and specificity of spectra. By pursuing these directions, future QP^2^‐SRS microscope will demonstrate greater potential in various areas such as molecular structure analysis, materials science, and biomedical imaging.

## Supporting Information

The authors have cited additional references within Supporting Information.^[^ ^55^ ^]^

## Conflict of Interests

The authors declare no conflict of interest.

## Supporting information



Supporting Information

Video S1

Video S2

Video S3

## Data Availability

The data that support the findings of this study are available in Supporting Information of this article.
