# Supplementary material for: High‐Specificity Spatiotemporal Cholesterol Detection by Quadrature Phase‐Shifted Polarization Stimulated Raman Imaging
Source: Angew Chem Int Ed Engl. 2025 Jun 8;64(32):e202505038. doi: 10.1002/anie.202505038 (PMC12322629; doi:10.1002/anie.202505038)
Supplement: Supplementary file 1 — Supporting Information [file ANIE-64-e202505038-s004.pdf]

Supporting information

High-Specificity Spatiotemporal Cholesterol Detection by Quadrature Phase-Shifted Polarization Stimulated Raman Imaging

Yongqing Zhang, Xinyu Deng, Siming Wang, Wenyu Zhou, Zhengyan Wu, Xiaobin Tang, Hyeon Jeong Lee*, Delong Zhang*

*Corresponding author. dlzhang@zju.edu.cn, hjlee@zju.edu.cn

This PDF file includes:

Experimental methods
Figs. S1 to S13
Legends for Video S1 to S3

Other Supplementary Material for this manuscript includes the following:

Video S1 to S3

Experimental methods

The principle of detection of dual-polarization information for QP²-SRS microscope. The generation of SRS signal belongs to a third-order nonlinear optical process, in which the incident electric field interacts with the charge of the medium, resulting in nonlinear polarization $P^{(3)}$. Using Cartesian coordinate system, the polarization can be expressed as:

$$P^{(3)}(\omega_p) = \varepsilon_0 \frac{D}{4} \chi^{(3)}(\omega_p \, ; \, \omega_s \, , \, \omega_P \, , \, \omega_s) E_s E_P E_S^*$$

where ε_0 is the vacuum dielectric constant, $D = 3$ for $\omega_0 = \omega_P$ or ω_s. E is the complex amplitude of the electric field, $\chi^{(3)}$ is a four-classes tensor of 81 components describing SRS, and for isotropic media, the magnetic susceptibility $\chi^{(3)}$ has 21 non-vanishing elements, of which only three of them are independent [55]. When we take the electric field direction of E_P as a reference and write it as 1, it is worth noting that $Im[\chi^{(3)}_{1111}]$ corresponds to $I_\parallel$ in the spontaneous Raman measurement and $Im[\chi^{(3)}_{1221}]$ corresponds to $I_\perp$ in the spontaneous Raman measurement. This means that by changing the polarization angles of the Stokes and pump fields in the SRS process, we can obtain the same parallel and perpendicular spectral information as spontaneous Raman scattering. Specifically, the SRL signal field caused by excited Raman emission under molecular vibration can be expressed as:

For parallel polarization, $E_{SRL,\parallel}(\omega_p) = i\,\chi^{(3)}_{1111} E_{s1} E_P E^*_{s1}$,

For perpendicular polarization, $E_{SRL,\perp}(\omega_p) = i\,\chi^{(3)}_{1221} E_{s2} E_P E^*_{s2}$

the intensity of the optical signal reaching the pump on PD is composed of the incident field at frequency E_P and the field generated by stimulated Raman effect E_{SRL}:

$$I_{total} = |E_P + E_{SRL}|^2$$

when the signal field is weak relative to the local oscillator ($E_P \gg E_{SRL}$), what is actually detected is the intensity change of the pump field, i.e., the SRL signal is the interference term between the two fields and is extracted by the lock-in amplifier through heterodyne detection. For parallel and perpendicular cases, the actual SRL signal strength is as follows:

For parallel polarization, $I_{SRL,\parallel}(\omega_p) = Im[\chi^{(3)}_{1111}] I_P I_{s1}$,

For perpendicular polarization, $I_{SRL,\perp}(\omega_p) = Im[\chi^{(3)}_{1221}] I_P I_{s2}$

when irradiating the sample with a Stokes beam that contains both parallel and perpendicular components, differing by a quarter-period time delay, along with the pump beam, the signal composition $I_{SRL}(\omega_p, t)$ can be expressed in the time domain as follows:

$$I_{SRL}(\omega_p, t) = I_{SRL,\parallel}(\omega_p) \sin\left(\frac{\pi f_0 t}{2} + \phi_0\right) + I_{SRL,\perp}(\omega_p) \cos\left(\frac{\pi f_0 t}{2} + \phi_0\right)$$

where, f_0 is the repetition frequency. Thus, the parallel and perpendicular spectral information can be extracted synchronously with the multichannel lock-in amplifier by selecting the appropriate initial phase ϕ_0 and generated in two channels orthogonal to each other without crosstalk.

QP²-SRS microscope setup. Based on a traditional SRS imaging system, ps laser (picoEmerald, Applied Physics & Electronics) provides a wavelength tunable pump beam (700 – 990 nm) and a fixed wavelength Stokes beam (1031 nm) with a repetition frequency of 80 MHz and a pulse width of 2 ps. The Stokes beam is modulated by the EOM to form a repetition frequency of 20 MHz. The HWP was used to adjust the Stokes beam after the EOM (EO-AM-NR-C2, Thorlabs) to a 0°, 45°, or 90° linearly polarized beam compared with the pump beam. In contrast to the previous method, we separate the parallel and perpendicular components of a 45° linearly polarized Stokes beam, and introduce a time delay with a $\pi / 2$ phase difference between the two separated Stokes beams. The two beams were then combined using another PBS. In this way, the Stokes beam contains both parallel and perpendicular

components but differs in time by a quarter of a cycle. Then, the SRL signal is obtained by spatiotemporal focusing of the pump and Stokes beams onto the sample. When the Stokes beam is filtered out by a low-pass filter, the SRL signal contains both parallel and perpendicular polarization information in the pump beam, but the phase difference between the two in the time domain is fixed at $\pi / 2$. The parallel and perpendicular SRL signals are then distributed into two orthogonal channels via a multichannel lock-in amplifier (HF2LI, Zurich Instruments). We are able to extract these two fixed-phase signals sequentially and synchronously, thus achieving QP²-SRS imaging of a single PD with a dual-polarization signal (Fig. 1F).

QP²-SRS spectra acquisition. The acquisition of QP²-SRS spectrum is realized by the wavelength sweeping function of ps laser. QP²-SRS spectra of pure substances are all from hyperspectral imaging of pure substances, and then they are extracted for subsequent analysis and processing. For TAG and cholesterol spectra acquisition, dwell time of single point is 20 μs, pump power is 100 mW, Stokes perpendicular-polarization component is 200 mW, parallel-polarization component is 200 mW. For the other three materials, the spectra obtained the parameters of a single point dwell time is 20 μs, the pump light power is 200 mW, the perpendicular-polarization component of Stokes is 300 mW, and the parallel-polarization component is 150 mW. The mixed solutions of TAG and cholesterol are dissolved in deuterated chloroform, and the spectral parameters obtained are consistent with the former. Here, all spectra are normalized for power and intensity.

HEK cell imaging. Serum-free Dulbecco's modified Eagle's medium (DMEM, Thermo Fisher) medium containing 5mM Methyl-β-cyclodextrin (MβCD, Aladdin) was used to conduct cholesterol depletion experiment in HEK 293 cells. Cells were seeded in 35 mm glass-bottom dishes and cultured with DMEM containing 10% fetal bovine serum (FBS) and 1% penicillin/streptomycin (P/S). The glass-bottom dishes were treated with 1mL cell adherent reagent (Applygen Technologies Inc., diluted in PBS buffer at a ratio of 4:500) for half an hour in advance to ensure normal adherent of the cells. After attached to the wall overnight, cells were treated with MβCD solution for 2h, washed with serum-free medium for three times, and then cultured with serum-free medium for 24 hours. Lastly, the cells were fixed with 4% Paraformaldehyde Fix Solution (PFA Fix Solution). For cell imaging, dwell time of single point is 20 μs, pixel size is 150 nm, pump power is 100 mW, stokes perpendicular-polarization component is 300 mW, parallel-polarization component is 200 mW.

Seminiferous tubule imaging. Ten-week-old male C57BL/6 mice (Shanghai Model Organisms Center, Inc.) were used in this study. Mice were euthanized via inhalation of 5% isoflurane overdose, followed by cervical dislocation to confirm death. All euthanasia procedures adhered to institutional guidelines for animal care and use and were approved by the Animal Care and Use Committee of The Third Affiliated Hospital, Guangzhou Medical University. Testes were collected, embedded in OCT compound, and processed into frozen sections for subsequent experiments. QP²-SRS imaging at perpendicular configuration was performed with pump power of 80mW and Stokes power of 100mW. The dwell time of SRS images is 20 μs and pixel size is 375 nm.

Liver tissue imaging. Liver tissue was procured from a mouse model of non-alcoholic steatohepatitis (NASH), which was created by administering a single low dose of streptozotocin at 2 weeks of age to induce insulin resistance, followed by a high-fat diet beginning at 8 weeks of age. The entire lifespan for the mice is approximately 32 weeks during which steatohepatitis and NASH is expected to develop progressively over time. In this study, 4 mice were generated and sacrificed at 2 time points with data recorded with 1 section per mice. Among these groups, the Ctrl (control) and high-fat diet (HFD) samples were obtained from mice that were 16 weeks old, while the ND (control) and FPC samples were collected from mice that were 32 weeks old. The four liver tissues were collected and snap-

frozen in liquid nitrogen. Approximately 7 μm sections of the livers were cut using a cryostat, placed on glass slides, and stored at −70 °C. Perpendicular-polarization hyperspectral SRS imaging was performed with 100 mW pump power and 200 mW Stokes power. The dwell time of SRS images is 20 μs and pixel size is 225 nm.

***C. elegans* imaging.** *C. elegans* are provided from Wei Zou lab in Zhejiang University (Institute of Translational Medicine of Zhejiang University). After a 0.8mM anesthetic was administered to make movements insignificant, living *C. elegans* are sandwiched with two 170-μm cover glasses. Dual-polarization SRS imaging is performed at 2896 cm^{-1} and pump power is 100 mW, stokes perpendicular-polarization component is 150 mW, parallel-polarization component is 100 mW.

Davies-Bouldin Index (DBI). The Davies-Bouldin Index (DBI) is a metric used to evaluate the quality of clustering, where a lower value indicates better clustering performance. The DBI of two clusters is defined as follows:

$$DBI = \frac{1}{n}\sum_{i=1}^{n} max_{j\neq i}\left(\frac{S_i + S_j}{d(c_i, c_j)}\right)$$

where, S_i represents the average distance between each point in cluster i and its centroid, reflecting the dispersion of the cluster. $d(c_i, c_j)$ denotes the Euclidean distance between the centroids of clusters i and j.

Image analysis. All processes take place in the ImagJ software. All images are analyzed by power normalization first. For ratiometric analysis, each image was firstly subtracted with background and normalized, then added with a constant (with value = 1) to avoid abnormal values in divisions near zero. For ratiometric image of cells, in order to avoid interference of nucleus position on cholesterol quantification in LDs, non-lipid background was removed after nucleus position was determined by LASSO [19a]. The LDs average spectrum is obtained by determining the position of the LD through LASSO algorithm, and then extracting the SRS spectrum of each LD, and finally obtaining the average spectrum. To obtain the image of various components of spermatogenic tubules, livers, and *C. elegans,* BM4D is performed on the initial hyperspectral data, and the distribution map of each component is obtained by LASSO for Fig. 4, 5.

Statistical analysis. Droplets with a circularity from 0.6 to 1.0 were extracted using a minimal size threshold, determined by the resolution limit of the SRS system. The size and intensity ratio values were then measured. For analysis of the data, a two-sample t assay was used to determine significant differences (*, $p < 0.05$. **, $p < 0.01$. ***, $p < 1 \times 10^{-3}$. ****, $p < 1 \times 10^{-4}$).

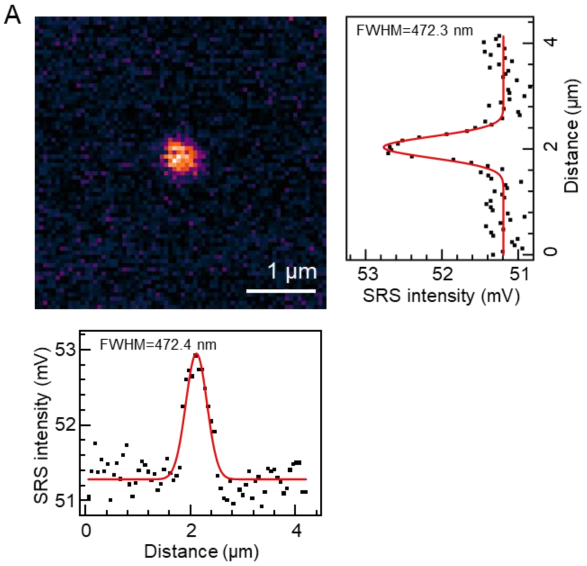

Fig. S1. The spatial resolution of QP²-SRS microscopy. (A) A single 200-nm PMMA bead imaging at 2957 cm^{-1}. The X- and Y-resolutions were from FWHM of profiles of a PMMA bead. Scale bar stands for 1 μm.

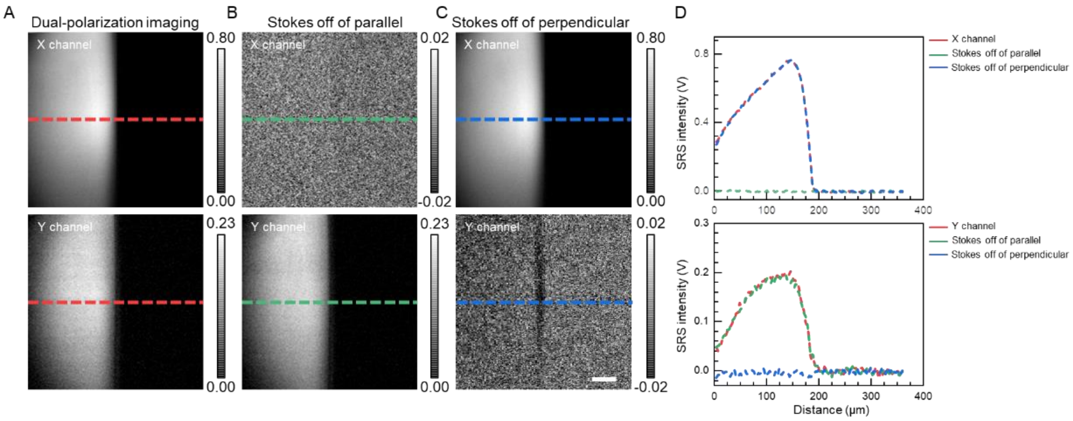

Fig. S2. Oil film imaging of QP²-SRS microscopy. (A) QP²-SRS imaging of oil film at 2896 cm^{-1}. (B) QP²-SRS imaging of oil film at 2896 cm^{-1} when parallel-polarization Stokes beam was off. (C) QP²-SRS imaging of oil film at 2896 cm^{-1} when perpendicular-polarization Stokes beam was off. (D) profiles of QP²-SRS imaging of oil film indicated by dashed lines in A-C. Scale bar stands for 50 μm.

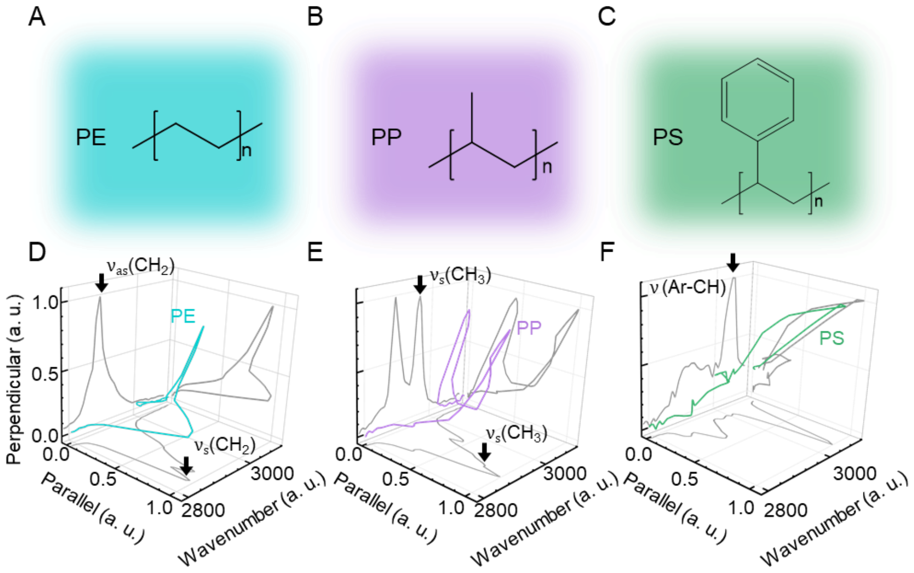

Fig. S3. QP²-SRS spectra of polymer. (A-C) Molecular structures of different polymers. (D-F) SRS spectra of different polymers in QP²-SRS.

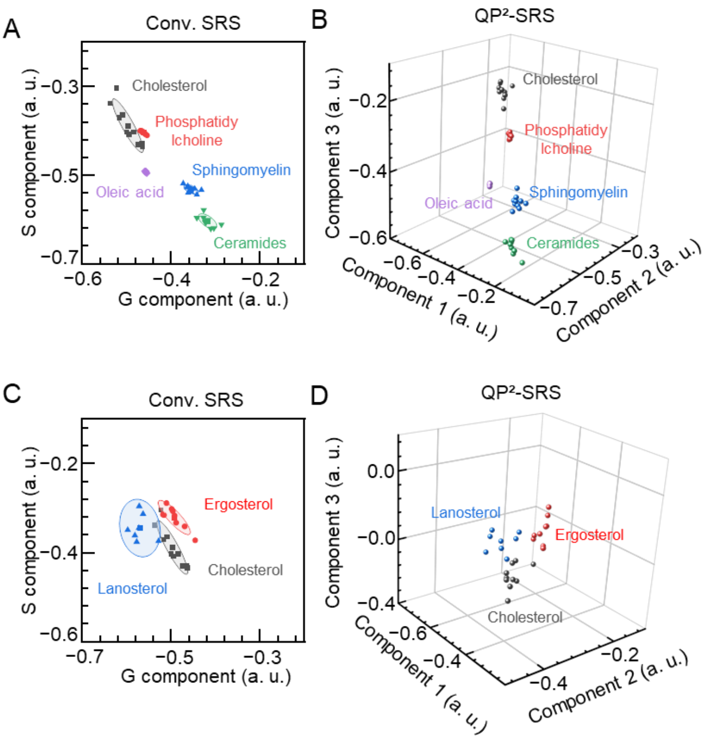

Fig. S4. Phasor plots of SRS spectra of different biomolecules. (A-B) Phasor plots of SRS spectra of different lipids including conventional SRS (A) and QP²-SRS (B). (C-D) Phasor plots of QP²-SRS spectra of similar sterol molecules in conventional SRS (C) and QP²-SRS (D).

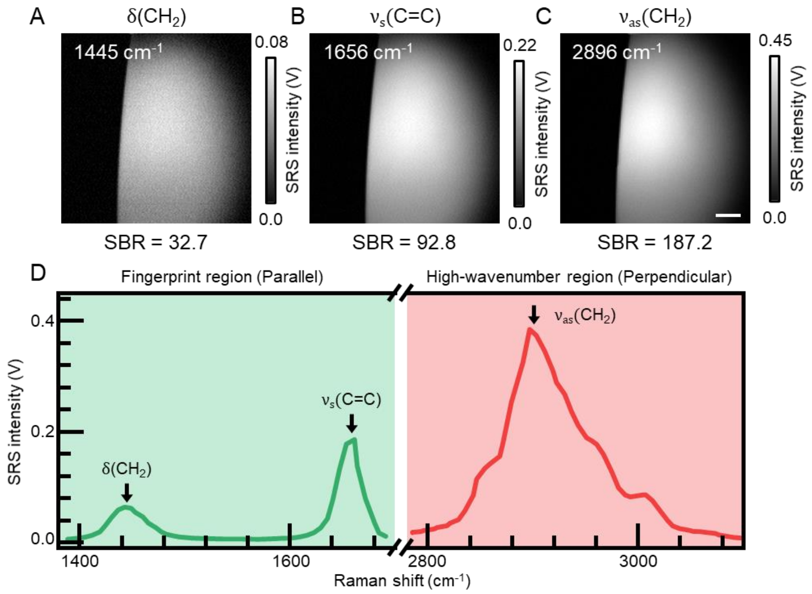

Fig. S5. SRS imaging of olive oil film at different Raman regions. (A) SRS spectra oil film at fingerprint region in parallel configuration and high-wavenumber region in perpendicular configuration by QP²-SRS imaging. (B-D) Oil film imaging at 1445 cm^{-1} (B), 1656 cm^{-1} (C) in parallel configuration and at 2896 cm^{-1} in perpendicular configuration (D) indicated by spectra A. Scale bar, 50 μm.

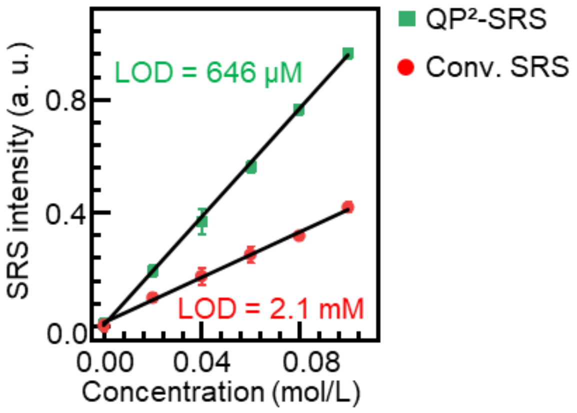

Fig. S6. Concentration dependence of signals. Lines: Linear fitting of SRS intensity at 2960 cm^{-1} of cholesterol solutions with different concentrations.

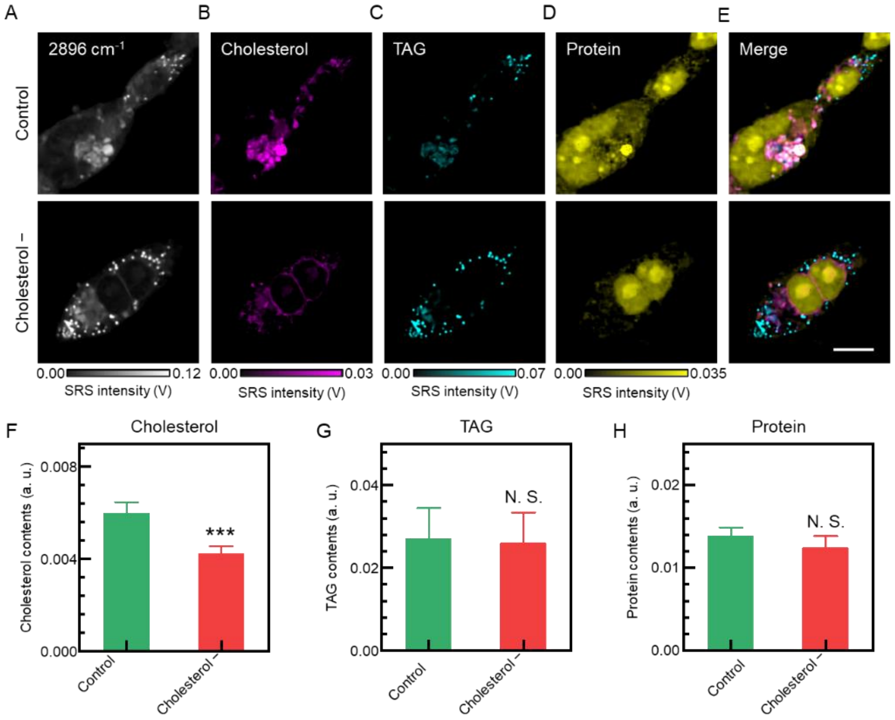

Fig. S7. Component analysis of hyperspectral QP²-SRS imaging of HEK cells. (A) QP²-SRS imaging of HEK cells at 2896 cm^{-1} in perpendicular configuration. (B-D) Localizations of cholesterol (B), TAG (C), and protein (D) obtained with LASSO analysis. (E) Merge images of different biomolecules from B-D. (F-H) Statistical analysis of biomolecules content of total cholesterol (F), TAG (J), and protein (H) in HEK cells. Scale bar, 10 μm.

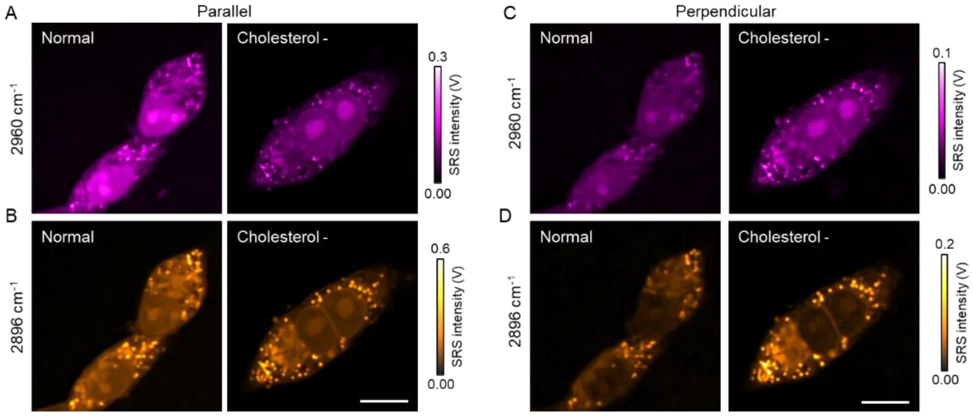

Fig. S8. QP²-SRS images of HEK cells. (A-B) HEK cells imaging at 2960 cm^{-1} (A) and 2896 cm^{-1} (B) in parallel configuration. (C-D) HEK cells imaging at 2960 cm^{-1} (C) and 2896 cm^{-1} (D) in perpendicular configuration. Scale bars stand for 10 μm.

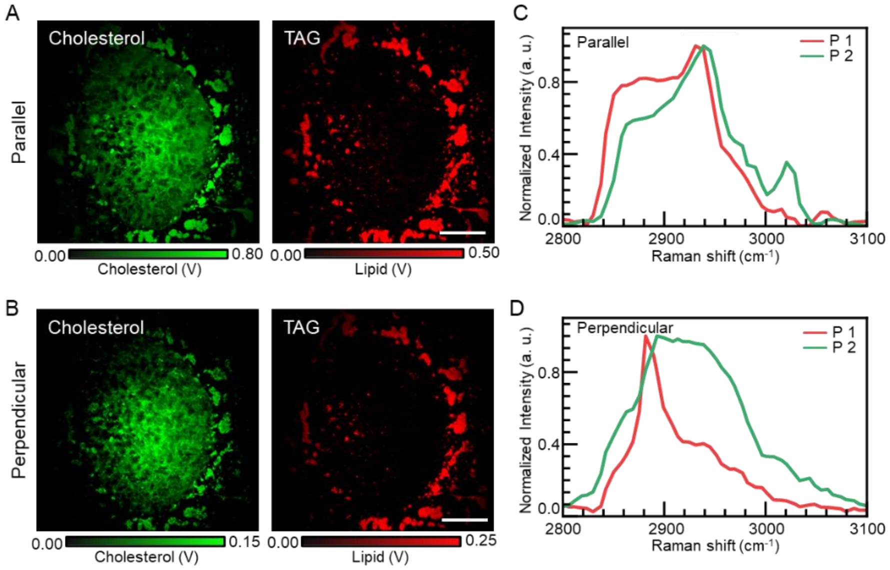

Fig. S9. Components of seminiferous tubules obtained by LASSO algorithm. (A-B) Cholesterol and TAG components of seminiferous tubules obtained in parallel (A) and in perpendicular (B) configurations. (C-D) QP²-SRS spectra of two LDs in parallel (C) and in perpendicular (D) configurations. Scale bars stand for 50 µm.

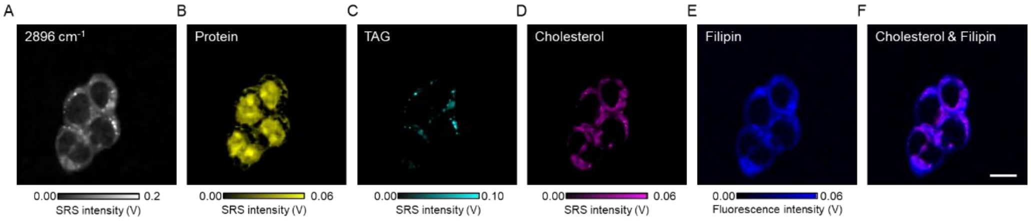

Fig. S10. Validation of cholesterol mapping in HEK cells. (A) QP²-SRS imaging at 2896 cm^{-1} of cell. (B-D) Localizations of different biomolecules obtained by QP²-SRS imaging. (E) Localizations of cholesterol with filipin III dyeing obtained by two-photon excited fluorescence. (F) Merge channels of cholesterol localizations obtained by QP²-SRS imaging and fluorescence imaging. Scale bar, 10 μm.

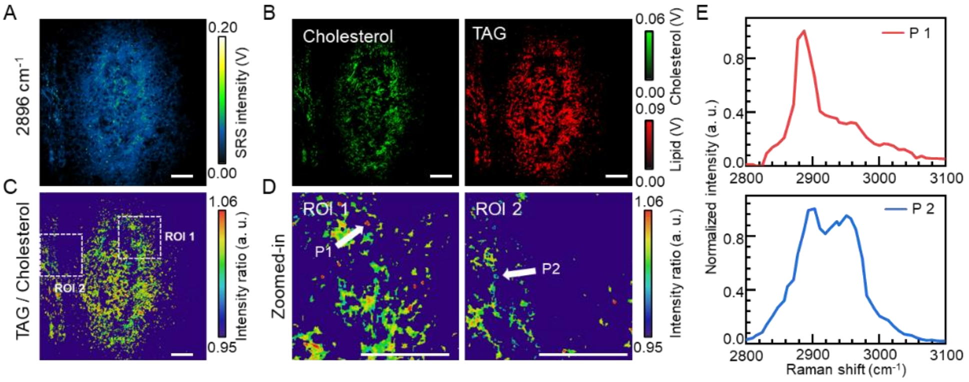

Fig. S11. Analysis of spatial distribution of lipids in seminiferous tubules. (A) QP²-SRS imaging of seminiferous tubules at 2896 cm^{-1}. (B) Cholesterol and TAG components of seminiferous tubules obtained by LASSO algorithm. (C) Spatial lipid component distribution mapping obtained by LASSO analysis of seminiferous tubules. (D) ROIs indicated by dashed boxes in C. (E) QP²-SRS spectra of different types of LDs indicated by arrows in D. Scale bars stand for 25 μm.

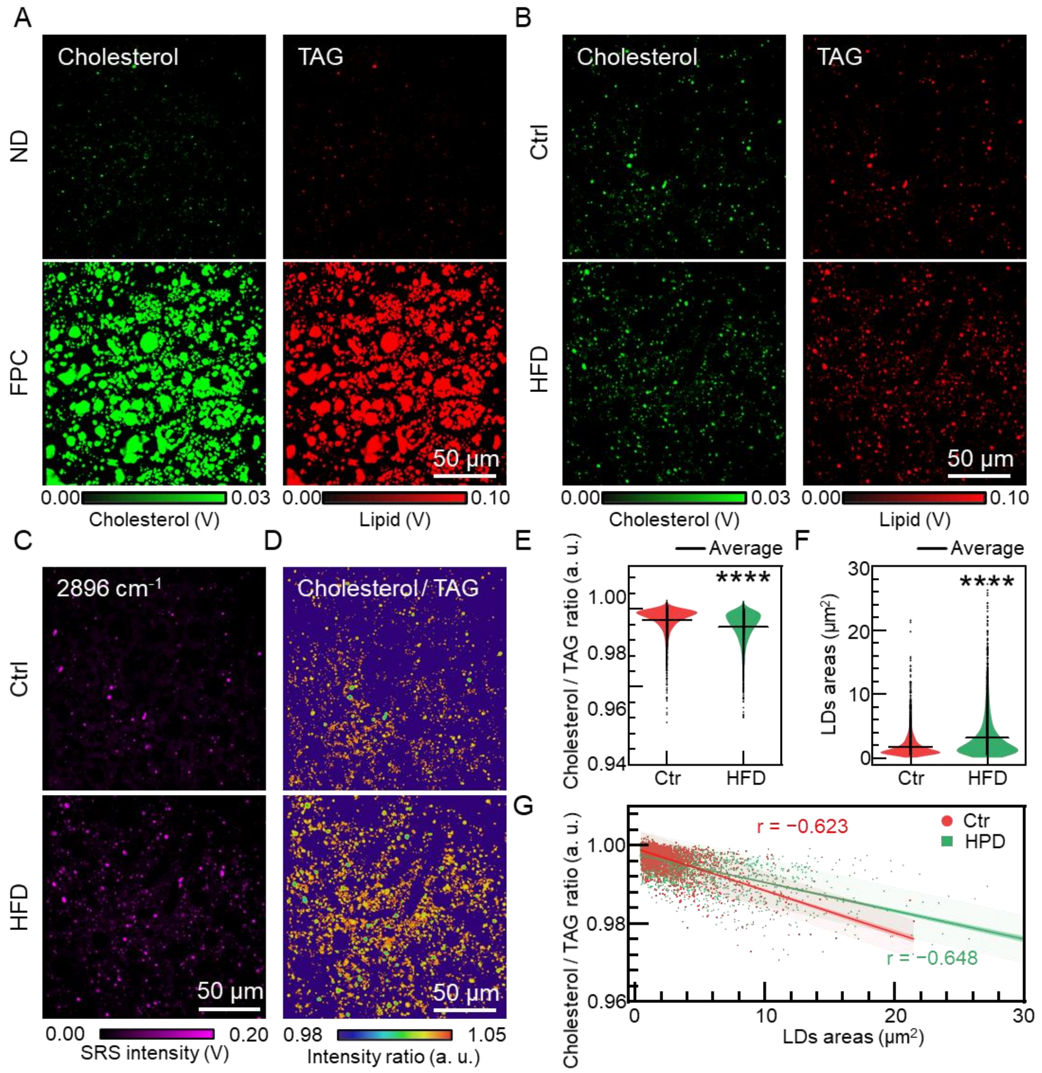

Fig. S12. Components of mouse livers tissue obtained by LASSO algorithm. (A-B) Cholesterol and TAG components of liver tissues in 32-week-old mice (A, ND and FPC groups) and 16-week-old mice (B, Ctrl and HFD groups). (C) SRS imaging at 2896 cm^{-1} of liver tissues in 16-week-old mice of Ctrl and HFD group. (D) Spatial lipid component distribution mapping obtained by LASSO analysis of liver tissues. (E) Lipid component analysis of LDs in different groups of liver tissues. (F) Average area of LDs changing in Ctrl and HFD groups. (G) Correlation between size of LD aggregates and cholesterol/TAG ratio of different groups. *, $p < 0.05$, **, $p < 0.01$, ***, $p < 0.001$, ****, $p < 0.0001$. n = 3 for both groups.

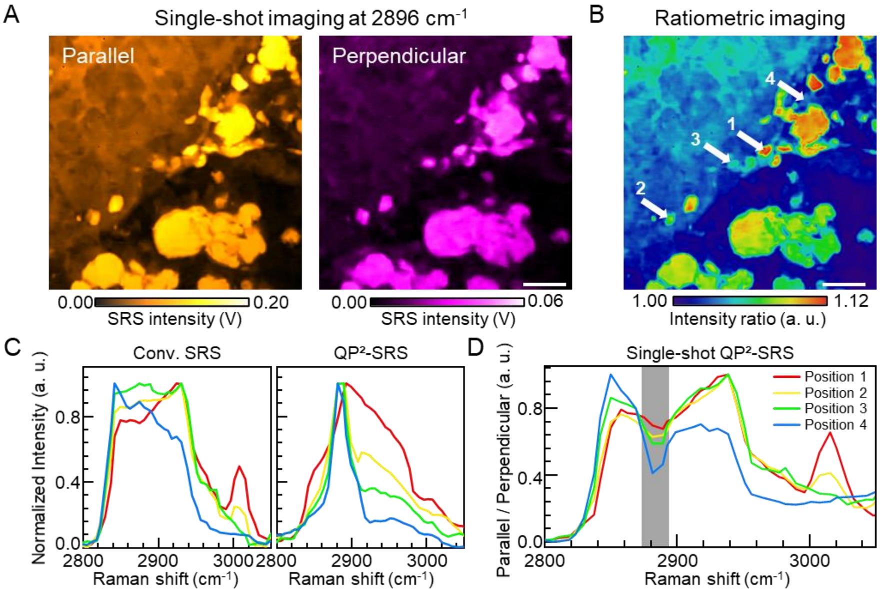

Fig. S13. Dual-polarization QP²-SRS imaging of seminiferous tubules. (A) Single-shot QP²-SRS imaging of lipid structures at 2896 cm^{-1} in seminiferous tubules. (B) Ratiometric QP²-SRS image of lipid structures obtained from A. (C) QP²-SRS spectra of different positions indicated by arrows in B in parallel (Left) and perpendicular (Right) configurations. (D) Ratiometric QP²-SRS spectra of different lipid structures indicated by arrows in B. Scale bars stand for 10 µm.

Video S1. 3D plots of biomolecules by spectral phasor analysis.

Video S2. A single LD dynamic imaging inside living *C. elegans*. This video file shows dynamic images of fat droplets in 6-second intervals over approximate 9 minutes. Each frame, consisting of 80 by 80 pixels, is acquired with a pixel dwell time of 5 µs.

Video S3. Dynamic imaging of discrete LDs aggregation into LDs clusters inside epithelium of living *C. elegans*. This video file shows dynamic images of fat droplets in 6-second intervals over approximate 9 minutes. Each frame with 110 by 375 pixels is acquired with pixel dwell time of 5 µs.
