# Supplementary figures and images for: High‐Specificity Spatiotemporal Cholesterol Detection by Quadrature Phase‐Shifted Polarization Stimulated Raman Imaging
Source: Angew Chem Int Ed Engl. 2025 Jun 8;64(32):e202505038. doi: 10.1002/anie.202505038 (PMC12322629; doi:10.1002/anie.202505038)

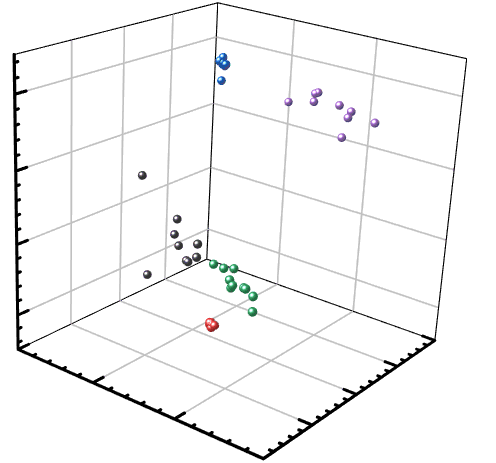

Supplement: Supplementary file 2 — Video S1 [file ANIE-64-e202505038-s001.gif]

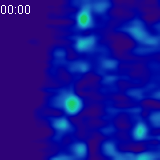

Supplement: Supplementary file 3 — Video S2 [file ANIE-64-e202505038-s002.gif]

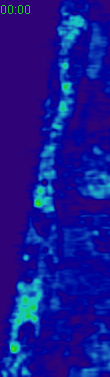

Supplement: Supplementary file 4 — Video S3 [file ANIE-64-e202505038-s003.gif]
